# Diseases of the Heart and Circulation

**Published:** 1902-12-27

**Authors:** 


### Diseases of the Heart and Circulation.

(*Concluded from page 197.*)

**Inequality of the Pupils in Aortic Aneurysm.**—Wall and Walker [5] have recently offered a new explanation of this well-known fact, which has hitherto been thought to be due to interference with the sympathetic. They state that this theory is unsatisfactory: 1, on anatomical grounds, because in the majority of cases there is no evidence of implication of that portion of the sympathetic nerve trunk containing pupil dilator fibres, and because it is not established that sympathetic filaments supply the sac wall of an aneurysm; 2, on physiological grounds; because the explanation supposes that the same conditions may produce sometimes irritation and sometimes paralysis of the nerve; because cases are rare in which there is evidence apart from the pupillary change that the sympathetic is involved; and because the pupillary changes are not those met with in cases where the sympathetic is involved. Alterations in vascular conditions may be associated with alterations in the size of the pupils; thus high arterial tension is associated with small pupils, and low arterial tension with large ones. The cause of this is probably to be found in the spiral structure of the vessels of the iris. Local inequalities of blood pressure may therefore be associated with inequalities of the pupils; thus, enlargement of the pupil is frequently associated with diminution of the temporal and radial pulses on the same side of the body; and obstruction of the carotid artery on one side of the neck is associated with enlargement of the pupil on the same side. Similarly digital compression of the carotid artery will cause enlargement of the pupil on the same side. The authors conclude that inequality of pupils associated with thoracic aneurysm is usually due to inequality of blood pressure in the ophthalmic arteries resulting from the abnormal vascular conditions.

**Intermittent Claudication.**—Walton and Paul [6] suggest the name angina cruris for this disease, which was first described by Charcot in 1858, for the essential symptom is intense paroxysmal pain, whilst the lameness is by no means a constant feature. The painful paroxysms are probably of vascular origin, and result from vascular spasm, perhaps with increased blood-pressure acting on vessels partly occluded whether from local or general disease (aneurysm, syphilis), from senile changes (atheroma), or from congenital tendency to angiofibrosis. The occurrence of the paroxysmal pains with pulseless pedal arteries is too constant to be explained by coincidence, though pulseless arteries may be found without the pains, and conversely The authors think that many cases of recurring local cramps properly belong to this condition, particularly when associated with vascular changes.

**Sclerosis of the Superficial Veins.**—Sonques and Jauvier [7] have described a case of this very rare condition. A man, aged 40, who had not suffered from syphilis, but was addicted to alcohol, came under observation for incipient pulmonary tuberculosis. The superficial veins of the limbs were indurated, smaller than normal, hard and resistant, and rolled under the fingers like a cord. The induration had come on gradually and the patient was not aware of its existence, nor was he affected thereby. The radial arteries were hard, but the temporal arteries were not tortuous. The heart and kidneys were normal. The lesions differ from those of chronic phlebitis, the indurated vein is like a contracted artery, its lumen is scalloped and narrowed, and the folds formed by the intima are free from clots. Its pathology is obscure; some have suggested that it is similar to that of arterio sclerosis.

**Caisson Disease.**—Macmorran [8] has reported the cases of caisson disease which occurred during the contruction of the Greenwich footway tunnel, which occupied about thirteen months. During the whole of that time there were but nine cases. This small number was due to the ample supply of fresh air. Three of the cases were serious, the others slight, all of them had giddiness as a symptom, only one had pains in the joints, two had pains in the back and thighs, one had recurrent epistaxis, with pain in the nape of the head and forehead, and shortness of breath. It was found that the presence of carbonic acid gas in the air in undue proportions causes an increase in the number of cases of caisson disease. This suggests that under the increased pressure the interchange of gases in the lungs is interfered with, so that the $CO_2$ is less readily eliminated. To this we must add the hyperæmia of the deeper tissues due to the pressure, so that the circulation is retarded. Owing to these two causes, hyperæmia of the nerve centres and accumulated impurities in the blood, there is a double effect on the sympathetic and vasomotor systems, the consequences of which are not manifest till varying times after the cause has been removed, when reaction occurs manifested in the sensory and sympathethic nerves throughout the body. By far the best treatment is the medical lock, into which a purified air should be pumped till the pain goes, and then the pressure very gradually reduced. The air pumped in should be purified by passing it over an apparatus containing caustic soda in screens, so that most of the $CO_2$ is removed. Similarly the prevention of the worst form of caisson disease can be prevented by getting rid of the $CO_2$ where it collects in largest quantity, namely, in the shield where the men are working. This was done in the Greenwich tunnel, with the excellent results mentioned above. All men employed should be constantly examined, and should a booming sound in the heart or reduplication of the second sound be heard the man should be suspended altogether.

[5] Lancet, July 12. [6] Boston Med. and Surg. Jour., April 3. [7] Lancet, June 28. [8] Brit. Med. Jour., April 26.

